# COVID-19 economic stimulus packages, tourism industry and external debt: The influence of extreme poverty

**DOI:** 10.1371/journal.pone.0287384

**Published:** 2023-08-29

**Authors:** Luke Okafor, Usman Khalid

**Affiliations:** 1 School of Economics, University of Nottingham Malaysia, Semenyih, Selangor, Malaysia; 2 Department of Innovation in Government & Society, College of Business and Economics, United Arab Emirates University, Al Ain, United Arab Emirates; Bucharest University of Economic Studies: Academia de Studii Economice din Bucuresti, ROMANIA

## Abstract

The COVID-19 outbreak has had a catastrophic effect on the tourism sector and poverty alleviation efforts. This is especially the case, given the crucial role the tourism sector plays in poverty alleviation and the generation of foreign exchange earnings. This study investigates the moderating influence of extreme poverty on the underlying link between the size of the tourism industry and COVID-19 Economic Stimulus Packages (ESPs) while accounting for the influence of external debt. The results show that tourism-dependent economies with a larger share of individuals living in extreme poverty introduced larger ESPs to cushion the impacts of the COVID-19 outbreak. In addition, economies with larger external debt have less fiscal and monetary leeway to alleviate the negative effects of the COVID-19 outbreak.

## Introduction

The COVID-19 outbreak has led to economic downturns in most countries across the globe as well as an unparalleled loss of life and jobs. The economic consequences of the pandemic are expected to exceed those of the 2007–2009 Global Financial Crisis [1]. For instance, it is estimated that the disruptions resulting from the outbreak would lead to an increase in extreme poverty levels globally for the first time in 20 years, with around 150 million people falling into extreme poverty around 2021 [2].

The pandemic has disproportionately affected the tourism sector following domestic and global travel restrictions to dampen the spread of COVID-19. As a result, tourism-dependent countries implemented larger Economic Stimulus Packages (ESPs) to support the sector in an attempt to mitigate the economic damage from the outbreak [3]. The almost halt to international tourism flows has not only affected the revenue-generating ability of tourism-dependent countries, but it has also hampered poverty-reduction efforts. It has been well-established in the literature that expanding the tourism sector helps reduce poverty [4–6]. This suggests that extreme poverty is likely to play a moderating influence on the underlying link between the level of tourism sector dependency and ESPs. Countries with a larger share of people living in extreme poverty augmented by sizeable dependency on the tourism sector are more likely to introduce larger ESPs in the presence of the COVID-19 pandemic.

Furthermore, an important consideration when assessing the impact of the poverty-tourism nexus on policy responses to the pandemic is the influence of external debt. High levels of external debt may reduce a country’s capacity to implement aggressive ESPs to cushion the negative effects of the outbreak. This is because countries burdened with high external debt may have limited fiscal space and monetary leeway to introduce large ESPs to boost economic activity. This is analogous to the depressing effect of debt on growth and/or domestic savings [7, 8].

Against this backdrop, this study investigates whether extreme poverty has a moderating influence on the link between the level of tourism sector dependency and COVID-19 ESPs while accounting for the influence of external debt on the underlying relationship. More specifically, this study investigates whether tourism-dependent countries with a large share of individuals living in extreme poverty introduced more aggressive ESPs vis-à-vis tourism-dependent countries with a smaller share of individuals living in extreme poverty, given their levels of external debt.

The contributions of this study to the strand of the literature that deals with the COVID-19 pandemic and the tourism sector are fourfold. First, this is the first study to investigate the moderating influence of extreme poverty on the underlying relationship between the level of tourism sector dependency and the COVID-19 ESPs. Unlike studies in the extant literature that focused on issues, such as the connection between the size of the tourism sector or the level of a country’s resilience and COVID-19 ESPs (see, e.g., [3, 9]), the current study explores the role of extreme poverty on the underlying relationship. The results indicate that in the presence of a larger share of individuals living in extreme poverty, tourism-dependent economies introduced larger ESPs to mitigate the negative impacts of the COVID-19 outbreak. This underscores the need for policymakers to introduce poverty reduction policies, such as economic and institutional reforms, promoting microfinance, and related programs, among others. Policies that tackle extreme poverty will serve as a buffer for governments in the event of a health crisis, such as the COVID-19 pandemic. This is because the share of resources that would be devoted to mitigating the impact of a major health crisis would potentially be lower if the share of individuals that are extremely poor is low or negligible.

Second, this study contributes to the literature by investigating whether the external debt of a country affects the COVID-19 ESPs. The results show that economies with a larger share of external debts have little fiscal and monetary leeway to cushion the negative effects of the COVID-19 outbreak. This suggests that a high debt burden is potentially counterproductive during a major health crisis, such as the COVID-19 outbreak. This is because the capacity of governments to mitigate the negative impacts of a major health crisis is limited in the presence of a high level of external debt. This reinforces the need for effective debt management, especially in the tourism-dependent countries. A country with an unsustainable debt level would have little or no leeway to mitigate the negative impacts of catastrophic events, such as the COVID-19 pandemic.

Third, this study contributes to the literature by showing that the influences of GDP per capita and hospital beds are unlikely to be significant in the short run in the event of a major crisis such as the COVID-19 pandemic. This underscores the need for policymakers to allocate adequate resources towards building up capacity and resilience, such as putting in place an effective national emergency, preparedness, response, and recovery as similar catastrophic disasters may occur in the future. Fourth, this study contributes by articulating a conceptual model/theory that explains the potential links between the size of the tourism industry, external debt, and COVID-19 ESPs. This includes the moderating influence of extreme poverty on the underlying link between the size of the tourism industry and COVID-19 ESPs.

The remaining part of the paper is organized as follows. The theoretical background and an overview of the related literature are presented in the next section. Section 3 introduces the data used in the empirical analysis, followed by the methodology of the study in Section 4. Section 5 presents the discussion of the results of the empirical analysis, and the last section concludes the paper.

## Theoretical background and review of the literature

The COVID-19 pandemic is nothing short of an extreme health crisis that turned into an economic and social shock with wide-ranging consequences for the economic and social well-being of several countries. More specifically, the COVID-19 pandemic brought the world to a standstill, and it had adverse impacts on both the economic and social fronts due to its sudden emergence. However, due to its highly contagious nature, physical travel, and trade of services were disproportionately more affected and, as a result, were halted or severely curtailed. The impact on the tourism sector has been more pronounced, with the tourism sector’s direct GDP reduced by more than half in 2020 compared to 2019 [10].

The COVID-19 pandemic was similar in many ways to the previous multiple crises that had adverse impacts on the tourism industry. The effect of the COVID-19 outbreak on global tourism, however, has been unprecedented. The crisis management literature documents the importance of proactive, preventive, and reactive policies in response to a crisis [11, 12]. Nevertheless, the crisis management frameworks that relate to the tourism and hospitality sector seem to be recovery centric, underscoring the importance of reactive policies for the recovery of the sector [13, 14]. A typical reactive crisis management policy generally involves assistance from the government that takes the form of aid packages, tax breaks, prolonged firm credit, and greater funding for marketing [15–17].

In the context of the COVID-19 outbreak, several studies have highlighted the need for aggressive and reactive policies to dampen the effects of the outbreak on the tourism sector [18]. For instance, [19] highlight the importance of government intervention in boosting hotels’ support for their employees, which improves their job satisfaction and organizational commitment. Similar conclusions were reported by [20], indicating that wage subsidies as a form of government support is essential for firms’ survival and retention of employees.

At the macro level, [3] show that countries that are highly dependent on tourism introduce aggressive economic policies to dampen the impact of the COVID-19 pandemic. These policies include fiscal stimulus packages, monetary policies such as interest rate cuts, and macro-financial packages. Additionally, [9, 21] highlight the role of resilience and digitalization, respectively, on the underlying link between economic stimulus packages and the level of dependency on the tourism sector.

In view of the above, especially as it relates to the crisis management theories, countries with high dependence on the tourism sector tend to implement aggressive economic policies to mitigate the negative impacts of the COVID-19 outbreak on the tourism sector. Thus, we hypothesize the following:

*H1*: *Countries with greater dependence on tourism tend to introduce larger COVID-19 economic stimulus policies*.

While the dependence on the tourism industry potentially plays a key role in government policy response, as highlighted above, a key variable that is overlooked in the literature is the role of poverty on the underlying link between the level of dependency on the tourism sector and government policy response. The primary reason why poverty potentially moderates the link between the level of dependency on the tourism sector and government policy response is that the tourism sector can be inherently pro-poor for several reasons [22]. Primarily, because tourists physically visit the destination, the sector offers greater linkage with other local enterprises, particularly smaller ones that are unable to support an online presence [22]. This is significant because tourism is an export that does not face trade barriers like tariffs from developed countries, unlike other agricultural or manufacturing exports.

Furthermore, in developing countries with few alternative export options, tourism offers greater potential for growth, especially in the presence of resources like nature and culture around which the sector is developed [22]. Moreover, because the sector is likely to employ more women than other competing industries, such as agriculture and manufacturing, tourism can empower women and provide their families with greater spending power [22, 23]. Not only that, the tourism sector is also a labor-intensive sector and hence is the most suitable sector for creating jobs for marginalized populations [24]. Similarly, the tourism sector can provide alternative income-generating opportunities to the agriculture sector labor during their off seasons [25].

The direct link between tourism development and poverty reduction may depend on whether tourism development leads to more job creation. As highlighted earlier, the COVID-19 pandemic slowed economic activity across countries, resulting in greater poverty levels across countries. A World Bank estimate reports that the COVID-19 pandemic is likely to push around 150 million individuals into extreme levels of poverty [2]. In addition, the economic activity of tourism-dependent countries was severely reduced vis-à-vis non-tourism-dependent countries due to the almost complete halt of international tourism flows. Consequently, significant job losses were reported in the tourism sector. [26] estimated that due to a US$ 1.58 trillion loss in tourism revenues in 2020 across 132 countries, around 24 million direct tourism jobs were at risk. Given that the poverty alleviating role of tourism is mainly a result of job creation in the sector, poverty in countries with a large tourism sector likely increased substantially compared to other countries.

The potential increase in poverty levels resulting from the impact of the COVID-19 pandemic is in line with the evidence in the extant literature. For instance, [27] estimated the impact of the COVID-19 outbreak on poverty in Indonesia. Their findings indicate that under the best-case scenario, the COVID-19 pandemic is likely to push 1.3 million more people into poverty, primarily due to a decrease in travel and tourism. Similarly, for the case of Indonesia, [28] highlight that poverty levels increased more for destinations/regions with international tourist attractions compared to destinations/regions that are not dependent on tourism. Similar findings were reported by [26], indicating that tourism-dependent countries were exposed to substantially more job losses. In addition, workers dependent on the tourism sector became poorer vis-à-vis non-tourism workers within and across countries.

In response to increasing poverty levels due to the COVID-19 outbreak, several economies have introduced policies to reduce the impact of the pandemic on poverty. For example, in Indonesia, a pre-employment program was redesigned from a training program for employment for recent graduates and unemployed workers to offer cash assistance to those who lost their jobs due to the pandemic [29]. As most of the pandemic’s impact on poverty is observed via job loss, one way to tackle poverty is with fiscal and monetary benefits to businesses to help them retain their workers during an economic slump [30–32].

Governments in both developed and developing economies responded to the pandemic with varying policies or tailored policies to suit their country’s situation, coupled with aggressive fiscal policies to boost health expenditure, R&D, income transfers, and wage subsidies to firms to retain workers and dampen poverty levels [33]. Similarly, as highlighted by [34], countries in South Asia made a commitment to collectively tackle the issue of rising poverty levels due to the pandemic in the region by establishing a COVID-19 emergency fund (CEF). At the country level, each country formulated policies that aimed at helping poor and vulnerable groups, including migrants and informal sector workers that have little or no access to social safety nets [34].

Moreover, the Indian government announced a financial stimulus package that supported the poor by providing them with the assistance that includes some quantity of wheat or rice and some amount of pulses for every low-income household, some quantity of cooking gas cylinders for millions of poor households, a one-off cash transfer to millions of senior citizens, medical insurance for every front-line health employee, among others [35]. Similarly, in the case of China, the government introduced policies to support off-farm employment in rural areas, provided basic income to households who fell into poverty, and provided a six-month extension on the payment of their micro-credit loans [36].

The above discussion implies that tourism-dependent countries are more likely to experience a larger increase in poverty levels. This is also supported by anecdotal evidence. For instance, Timor-Leste, expected a 5% contraction of GDP due to COVID-19 and tourism losses, introduced a $142m stimulus package, accounting for about 10% of its GDP [37]. Compared to the 2008 Global Financial Crisis, the average stimulus packages of low-income countries were almost 260% larger, and 600% larger for lower-middle-income countries [38]. This is in sharp contrast to upper-middle-income and high-income countries, whose stimulus packages changed in size only by -26% and 13%, respectively [38].

In light of the above, it is expected that countries that rely heavily on tourism and also host a large share of the population that lives below the poverty line are likely to introduce aggressive economic policies to dampen the impact of a COVID-19 shock. Thus, we hypothesize the following:

*H2*: *Countries with greater dependence on tourism as well as having a larger share of their populations living in poverty*, *tend to introduce larger COVID-19 economic stimulus policies*.

The size of the economic stimulus policies introduced to support the economy is likely to be affected by a country’s level of external indebtedness. In general, with low external debts, stimulus packages can have a crowding-in effect for developing economies, in that, the policies help create jobs and boost economic growth [39]. In contrast, for countries without strong reserves and high debts, a larger share of government resources that could have been devoted to stimulus packages would be used for debt servicing. This implies that fewer resources are likely to be allocated to stimulus packages. This is less likely to yield immediate benefits to the poor, particularly in times like the Covid-19 pandemic, when urgent spending was required for social protection and welfare. If immediate poverty alleviation strategies are not maximized during a crisis, this could exacerbate poverty, income inequality, inflation, and decreased investor confidence.

[40] suggest that government debt was also more likely to affect stimulus packages during the pandemic because of the effects of the lockdown on the poorest segments of society. Poorest households were not only more vulnerable to death or severe illness from contracting COVID-19, but were also less likely to comply with lockdowns, given their dire need for an income. These segments are also those who gain the most from consumer stimulus policies. This means that developing economies facing high levels of debt had to strike a balance between implementing less strict lockdowns and transferring just enough cash payments to help sustain the poorer families. Overall, indebted economies were unable to provide enough funds to be able to fully support the poor without them needing to risk their lives and livelihoods for the sake of subsistence [40].

In general, of all countries most dependent on tourism, as measured by GDP contributions from the tourism industry, at least 73% are developing countries [41]. This suggests that poorer countries were hardest hit due to COVID-19’s effect on the tourism industry. Poorer countries generally have limited resources, weak institutions, greater vulnerability to shocks, and thereby larger amounts of public debt relative to advanced economies. These characteristics exacerbated the negative effects of the outbreak on poorer economies, and as such, required significant external funding for the fiscal initiatives needed to sustain their economies. For instance, in 2020, Jamaica’s extensive borrowing to finance its stimulus packages led to public debt levels crossing 108% of its GDP [42]. Overall, public debt relative to GDP increased by six percentage points after the pandemic for low-income developing economies [43]. This has left poorer countries more vulnerable to future shocks, with several, such as Sri Lanka, having defaulted on their foreign debts, primarily because of significant loss of tourism earnings [44]. In view of the above, it is expected that the size of ESPs will be dependent on the level of indebtedness of a country, and in particular, the external debt of the country. Therefore, we hypothesize the following:

*H3*: *Countries with higher external debt are likely to introduce smaller COVID-19 economic stimulus packages*.

**Fig 1:**
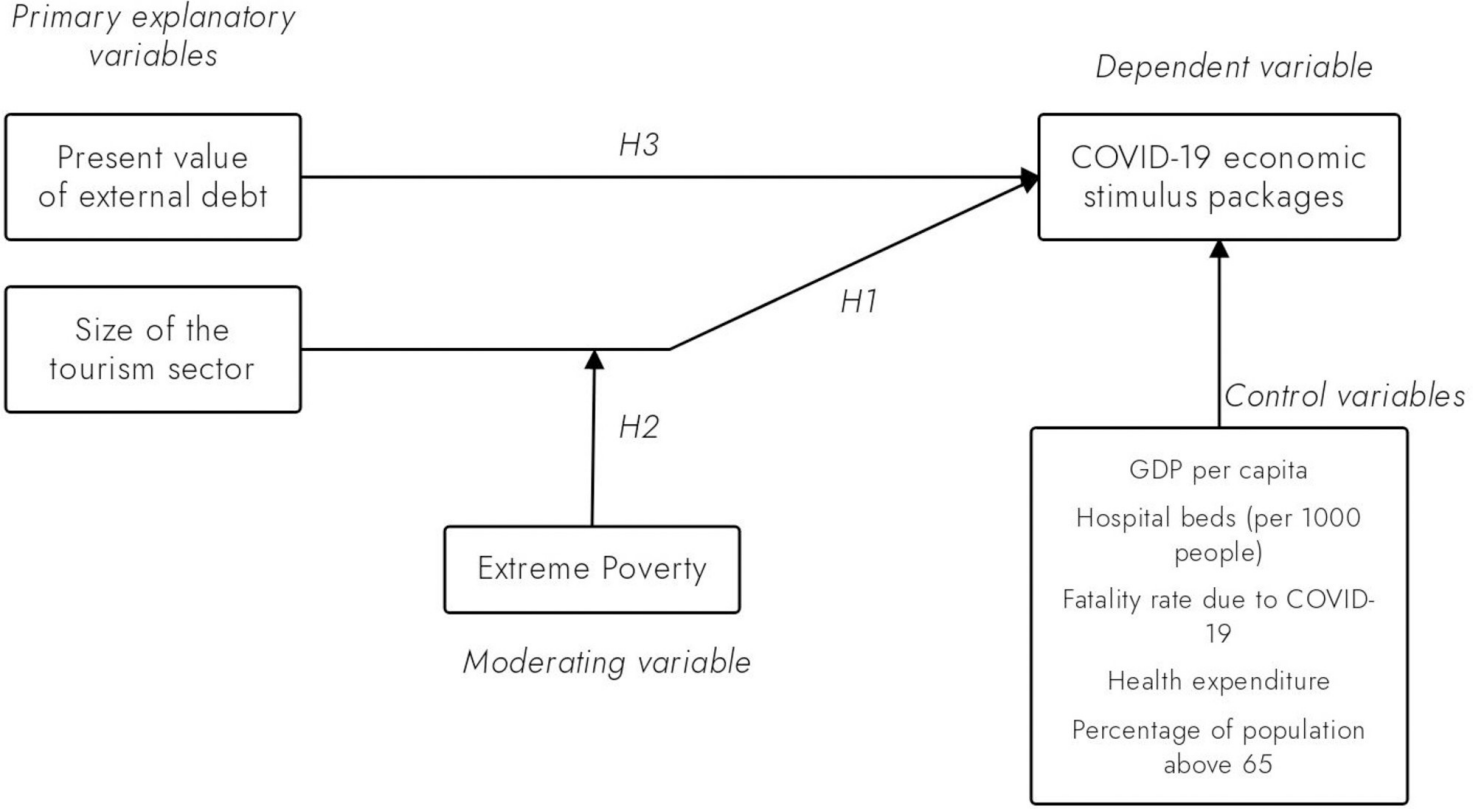
Conceptual framework.

The interrelationships between the COVID-19 stimulus packages, the size of the tourism sector, and external debt, as well as the moderating role of extreme poverty on the underlying link between COVID-19 stimulus packages and the size of the tourism sector, could be visualized using a conceptual model. For instance, Fig 1 presents the study’s conceptual model, showing that the size of the tourism sector tends to influence the COVID-19 ESPs (Hypothesis 1), while the level of extreme poverty in a country potentially moderates the link between the size of the tourism sector and COVID-19 ESPs (Hypothesis 2). In addition, the external debt of a country also potentially influences the size of the COVID-19 ESPs (Hypothesis 3). We also include a host of relevant control variables (discussed in the next section) that are likely to influence the COVID-19 ESPs.

While the hypotheses developed above serve as a guide for our empirical analysis, it is not without a caveat. Since the 1950s, the dominant view of the impact of tourism development on poverty alleviation has been based on ‘trickle-down’ and multiplier effects from its contribution to economic growth, in that those macro-economic benefits in the form of foreign exchange earnings, job creation, taxation, etc., would trickle down to the lower echelons of society [6]. Over time, this unmanaged, ‘automatic’ economic growth was found to marginalize the poor more than it helped uplift them.

Additionally, dependency theorists draw parallels between the tourism industry and colonialism, highlighting that multinational corporations, investors, and local elites develop, as well as profit from assets possessed by the society, especially the poor, such as their natural resources and culture [45]. Because the poorest countries do not have strong domestic markets for tourism and are, as such, highly dependent on international markets for tourism and tourism development, in practice, it is difficult to completely eliminate exploitative leakages such as foreigners reaping profits, and importing labor, technology, goods, and services [22]. The active pursuit of pro-poor tourism development strategies by governments and private enterprises, however, helps local communities retain economic benefits locally and uplift the poor directly. To the extent that tourism sector development involves specific measures for recruiting and training local workers and integrating with local industries such as agriculture, construction, and transport—instead of relying on foreign imports alone—jobs can be created at all skill levels, and across multiple industries [22]. This is important because although tourism exports make up a significant percentage of GDP for countries where the world’s poorest reside, direct benefits from tourism are unlikely to reach the poorest 10% of society, primarily due to a lack of skills, education, and connections.

Politics and public policy also influence the extent to which tourism development could affect the poor and their incomes. In the presence of any crisis, such as the COVID-19 outbreak, government’s stimulus packages are an important means to rapidly stimulate aggregate demand but must ensure that marginalized communities are protected. Depending on the type of package and how aid is distributed, stimulus policies can have both short- and long-term impacts on poverty [46].

## Methodology

To test the hypotheses developed in the previous section, we extend the econometric model used by [3] by incorporating extreme poverty, the interaction of extreme poverty, and the level of tourism sector dependency while accounting for the influence of external debt. The empirical model for the moderating influence of extreme poverty on the underlying connection between the level of tourism sector dependency and COVID-19 ESPs while accounting for the role of external debt can be specified as:

$${CESI}_{i}=\alpha_{0}+\alpha_{1}Ln\;{PVEXTD}_{i}+\alpha_{2}{TODUM}_{i}+\alpha_{3}{EXTPOV}_{i}+\alpha_{4}({TODUM}_{i}\;X\;{EXTPOV}_{i})+\alpha_{5}Ln\;{HOSB}_{i}+\alpha_{6}Ln\;{FARATE}_{i}+\alpha_{7}Ln\;{HEALTHEXP}_{i}+\alpha_{8}{POP65}_{i}+\alpha_{8}{Ln\;GDPK}_{i}+\varepsilon_{i}$$ (1)

where Ln is the natural logarithm, *CESI* is the COVID-19 economic stimulus index, *TODUM* is the tourism-dependent dummy, *PVEXTD* is the present value of the external debt percentage of GNI, and *EXTPOV* is the share of the population living in extreme poverty. The control variables include real GDP per capita (*Ln GDPK*), health expenditure as a percentage of GDP (Ln HEALTHEXP), population over 65 (POP65), fatality rate due to COVID-19 (Ln FARATE) and hospital beds per 1000 people (Ln HOSB).

A priori, we expect α_2_>0 because the COVID-19 pandemic likely affected the tourism sector disproportionately more than other sectors, and therefore, countries that are highly dependent on the tourism sector are more likely to introduce aggressive economic policies to dampen the adverse effects of the COVID-19 pandemic. We expect α_1_<0, because a higher level of external debt implies that a larger share of government resources must be devoted to debt servicing and/or repayment of loans, and thus, fewer resources would be available for stimulus packages.

Furthermore, we expect the coefficient of the interaction term between the tourism dependency indicator and the share of the population living in extreme poverty to be positive (α_4_>0). This suggests a presence of complementarity between the size of the tourism sector and the share of the population living in extreme poverty. Amidst high level of tourism dependency and a larger share of the population living in extreme poverty, countries are more likely to devote a larger share of resources to mitigate the adverse effects of the COVID-19 pandemic compared with those countries that are not (See Section 2 for a detailed discussion on why poverty potentially moderates the link between the level of dependency on the tourism sector and economic policy response).

The next section discusses the definitions of variables in more detail. To test the robustness of our results, we also use alternative measures of economic policy as our dependent variables, which are discussed in the next section.

### Data sources

The dataset consists of a cross-section of 54 countries. The selection of countries is dictated by data availability (the number of observations in some specifications could be less than 54, due to missing values for some key variables). The final data set is obtained after merging several datasets, as described below.

#### Dependent variables

The dependent variables include a COVID-19 economic stimulus index, *CESI*, monetary policy index, and fiscal policy. The variables used for generating the dependent variables are constructed by [47] to capture the COVID-19 ESPs introduced by most countries. The version of data used in this analysis is the 13th CESI update, October 2020, by [47]. Similar to [21], we use principal component analysis to derive the CESI and monetary policy index. The variables used for deriving the CESI include fiscal stimulus packages (percent of GDP), interest rate cut by the central bank (percent of the ongoing rate as of February 1^st^, 2020), reserve requirement, macro-financial packages (percent of GDP), and specific balance of payment measures (percent of GDP). The variables used in deriving the monetary policy index include interest rate cuts, reserve requirements, as well as macro-financial packages. Please see [3] for details about the data set.

#### Explanatory variables

The explanatory variables include a tourism-dependent dummy (*TODUM*), the present value of the external debt percentage of GNI (*PVEXTD*), and the share of the population living in extreme poverty *(EXTPOV)*. In addition, we include an interaction term between tourism-dependent dummy and extreme poverty (*TODUM X EXTPOV*). *TODUM* is set to 1 if the ratio of tourist arrivals in country *i* to total tourist arrivals for economies available in the sample is greater than the median value of the ratio of tourist arrivals to total tourist arrivals for economies available in the sample, and 0, otherwise. By construction, countries with values greater than the median rely more on the tourism industry compared to those with values less than the median. Tourist arrival data for the year 2018 and *PVEXTD* for the year 2019 are sourced from the World Development Indicators (WDI) [48]. Data for the share of the population living in extreme poverty are sourced from [49] (we used data for the most recent year available since 2010).

#### Control variables

In line with studies in the existing literature [3, 9], we controlled for relevant variables that are more likely to affect the magnitude of ESPs introduced by different economies to lessen the health and economic impact of the COVID-19 outbreak. The relevant variables consist of real GDP per capita, health expenditure (% of GDP), population over 65, and hospital beds per 1000 people. The data for these variables are available at the WDI [48] for the period 2018 or the most recent accessible data. Fatality rate, measured as the ratio of total deaths relative to total confirmed cases, is also controlled for. Data on fatality rate are sourced from [50, 51] (data are either for 23^rd^ or 24^th^ May 2020, depending on data availability).

#### Summary statistics

Preliminary evidence suggests that more tourism-dependent economies tend to introduce greater ESPs as proxied by CESI, monetary policy index, or fiscal policy than economies that are less tourism-dependent. Additionally, tourism-dependent economies are likely to have lower external debt and a lower share of the population living in extreme poverty (S1 Table in the supporting information reports the summary statistics).

Figures from the unconditional means indicate that more tourism-dependent economies tend to be richer, have more hospital beds, spend more on health, and have a larger percentage of the population over 65 than those that are not. Additionally, economies with higher dependency on the tourism industry are more likely to have a higher fatality rate than those that are not.

Figures from the correlation matrix suggest some evidence of multicollinearity issues involving GDP per capita and population over 65. This contrasts with the evidence from the tests of multicollinearity tests (See S2 and S3 Tables). Robustness checks would be conducted to see if the estimates are particularly sensitive to the exclusion of the affected variables.

## Discussion of results

The coefficients of the links between the tourism industry, external debt, and COVID-19 ESPs, as well as the influence of extreme poverty in the underlying relationships, are reported in Table 1. The indicator tourism sector dependency, *TODUM*, is positively related to different measures of economic policy response but statistically significant when CESI or fiscal policy is used as the dependent variable (see columns 1 to 6). This finding supports our first hypothesis and is consistent with the evidence in the extant literature that economies with higher tourism sector dependency implemented ESPs than those that are not [3]. In addition, extreme poverty is negatively associated with the monetary policy index (see columns 2 and 5).

**Table 1:** The links between the tourism sector, external debt, and economic policy response to the COVID-19 pandemic: The influence of extreme poverty.

	DV: CESI	DV: Monetary Policy Index	DV: Ln Fiscal Policy	DV: CESI	DV: Monetary Policy Index	DV: Ln Fiscal Policy
Variable	(1)	(2)	(3)	(4)	(5)	(6)
Ln PVEXTD	-0.179**	-0.226***	-0.190*	-0.158**	-0.205**	-0.155
	(0.075)	(0.081)	(0.109)	(0.078)	(0.082)	(0.119)
TODUM	0.524***	0.150	0.870***	0.392*	0.023	0.712***
	(0.185)	(0.173)	(0.243)	(0.219)	(0.203)	(0.259)
EXTPOV	-0.006	-0.014**	0.002	-0.007	-0.015**	0.0005
	(0.006)	(0.006)	(0.007)	(0.006)	(0.006)	(0.007)
TODUM X EXTPOV				0.027**	0.026**	0.034*
				(0.013)	(0.012)	(0.017)
Ln GDPK	0.074	0.025	-0.057	0.062	0.014	-0.072
	(0.154)	(0.116)	(0.226)	(0.155)	(0.115)	(0.223)
Ln HOSB	-0.078	-0.087	0.011	-0.063	-0.073	0.027
	(0.136)	(0.098)	(0.187)	(0.135)	(0.098)	(0.186)
Ln FARATE	-0.171*	-0.043	-0.363**	-0.164*	-0.037	-0.355***
	(0.095)	(0.089)	(0.136)	(0.094)	(0.090)	(0.131)
Ln HEALTHEXP	0.203	-0.088	0.454*	0.151	-0.138	0.387
	(0.329)	(0.340)	(0.244)	(0.344)	(0.350)	(0.242)
POP65	-0.027	-0.040*	-0.010	-0.022	-0.036	-0.003
	(0.029)	(0.023)	(0.037)	(0.029)	(0.024)	(0.035)
Constant	-1.725	0.388	-0.341	-1.590	0.517	-0.195
	(1.609)	(1.314)	(2.116)	(1.631)	(1.317)	(2.098)
Observations	54	54	52	54	54	52
R-Squared	0.363	0.326	0.372	0.391	0.351	0.398

Notes: DV denotes the dependent variable. Ln denotes natural logarithm, CESI is COVID-19 economic stimulus index, PVEXTD is the present value of external debt (% of GNI), TODUM is tourism-dependent dummy, EXTPOV is extreme poverty, GDPK is GDP per capita, HOSB is Hospital beds (per 1000 people), FARATE is fatality rate, HEALTHEXP refers to current health expenditure (% of GDP), POP65 is the percentage of population above 65. Robust standard errors in parentheses, levels of statistical significance indicated as

*** p < 0.01

** p < 0.05

* p < 0.10.

Interestingly, the joint impact of the tourism industry and extreme poverty is positive and statistically significant across different specifications (columns 4 to 6). This suggests that for a country that is tourism-dependent, an increase in extreme poverty level increases the size of the ESPs introduced by the country. In particular, for a tourism-dependent country, if the share of the population living in extreme poverty increases by one percentage point, then the size of the different measures of ESPs will increase between 0.026 and 0.034. This finding supports our second hypothesis suggesting that more tourism-dependent economies with a larger share of individuals living in extreme poverty introduced larger COVID-19 ESPs than those that are not.

We also find that external debt is negatively associated with different measures of economic policy response and is statistically significant except in one model specification (see columns 1 to 6). For instance, based on the coefficient reported in column 1, a one percent change in the present value of external debt reduces CESI by 0.0018. This suggests that economies with large external debt have less fiscal and monetary leeway to lessen the health and socio-economic impact of the COVID-19 outbreak, though the external debt is less responsive to fiscal policy intervention. This finding is in line with our third hypothesis. This is analogous to the dampening effect of debt on growth and/or domestic savings [7, 8].

Furthermore, there is no strong evidence that GDP per capita or hospital beds are associated with COVID-19 ESPs. These findings are in line with the evidence in the extant literature that also reports a statistically insignificant relationship between GDP per capita or hospital beds and COVID-19 ESPs [3, 9, 52, 53]. The evidence of no strong link between the two variables and COVID-19 ESPs can largely be explained in two ways. First, in the short run, a country’s health infrastructure, such as the number of hospital beds, is finite, which means that for a fast-spreading infectious disease like COVID-19, even having a better health infrastructure is unlikely to be of significant help. This is especially the case as the health infrastructure in most countries was not equipped to cope with a major health crisis, such as the COVID-19 pandemic. As a result, most countries are more likely to respond aggressively to such a shock regardless of the quality of health infrastructure. Second, the COVID-19 pandemic introduced a radical level of uncertainty across countries, especially at the beginning of the pandemic. As countries struggled to learn the best ways to respond to the COVID-19 pandemic, therefore, GDP per capita and health infrastructure were unlikely to play significant roles in alleviating the economic burden of the pandemic.

The fatality rate is negatively associated with CESI and fiscal policy (see columns 1 to 6). The impact of health expenditure is mixed, whereas the population over 65 is negatively associated with COVID-19 economic policy response, though mostly statistically insignificant. We tested the robustness of the coefficients by re-estimating the regressions, either excluding the GDP per capita or excluding both GDP per capita and the population over 65. The coefficients of variables of interest are fairly similar both in terms of sizes and signs (see S4 and S5 Tables).

## Conclusion

Findings from this study show that economies with higher tourism sector dependency augmented by a larger share of individuals living in extreme poverty implemented larger COVID-19 ESPs. In addition, economies with large external debt have less fiscal and monetary leeway to dampen the negative health and socio-economic effects of the COVID-19 crisis. Our results corroborate the findings in the existing literature that tourism-dependent countries are more likely to introduce aggressive economic policies to counter the negative impacts of the COVID-19 outbreak. However, our results also underscore the role of extreme poverty in the introduction of ESPs, adding an additional dimension to the findings in the existing literature.

Furthermore, our results call for the introduction of more targeted policies aimed at reducing poverty rather than policies that benefit only a few in society. Essentially, economic policies that consider the trade-offs between economic efficiency and equity are needed to fight against extreme poverty, especially in tourism-dependent economies. For instance, policies that reduce extreme poverty, such as means-tested benefits, including but not limited to income support, upskilling and reskilling programs, housing assistance, and family tax credits for vulnerable groups in society, would help to reduce the number of individuals that suffer from extreme poverty.

The findings from this study also highlight the importance of maintaining a healthy level of public debt, as it allows countries to introduce larger social welfare programs in the event of a health crisis, such as a COVID-19 pandemic. The capacity of a country, especially tourism-dependent economies, to strategically maintain a low-debt-to-GDP ratio on average is essential for building resilience against health crises, such as the COVID-19 outbreak. The maintenance of debt sustainability pays off during a major health crisis, as a government has more leeway in terms of the use of fiscal and monetary policies to mitigate the negative impacts of an exogenous shock, such as a COVID-19 pandemic.

Additionally, the findings of this study uncover that having a higher GDP per capita or a higher number of hospital beds is unlikely to play a significant role in the capacity of a country to mitigate the negative impacts of a major and unexpected health crisis in the short term. More specifically, amidst the radical uncertainty induced by the COVID-19 pandemic and inadequate national emergency preparedness, response, and recovery for a major health crisis in most countries, the role of GDP per capita in alleviating the economic burden of the crisis is unlikely to be substantial in the short-run. Likewise, given that most health facilities were ill-equipped and/or ill-prepared to simultaneously treat COVID-19 and/or non-COVID-19 patients during the health crisis, the role of hospital beds in reducing the economic burden of the crisis is also unlikely to be significant. This underscores the need for policymakers to devote adequate resources for building up capacity and resilience, especially robust national emergency preparedness, response, and recovery. This is essential as similar catastrophic events might occur in the future.

It is worthwhile to note that it could be argued that a high debt level can compromise the capacity of an economy to utilize ESPs to mitigate the negative effects of a health crisis, such as a COVID-19 pandemic, thus rendering the issue a moot point. This is not necessarily the case, as the insights gained from the findings of this study are important, especially in terms of the need for governments to embrace sustainable debt management. For instance, given the compromising effect of high debt levels, governments experiencing high debt levels can strategically reduce the debt levels by using options such as fiscal consolidation, growth-promoting policies, and taxing wealth, among others. This is because governments that manage their debts sustainably today will be better prepared to use fiscal and monetary policies in the future to combat negative health shocks, such as the COVID-19 pandemic.

It could be argued that the tourism businesses in many developing economies operate in the formal sector and thus are not captured in the regression model. We are unable to control for the potential influence of the informal sector on the underlying relationships due to data limitations. We acknowledge that this as one of the weaknesses of this study and an area for future study. Future researchers control for the size of the informal sector, subject to data availability, to see if the results of this study hold or not.

## Supporting information

S1 Table Summary statistics. (DOCX)

S2 Table Correlation matrix. (DOCX)

S3 Table Multicollinearity tests. (DOCX)

S4 Table The links between external debt, size of the tourism sector, and economic policy response to the COVID-19 pandemic: The influence of extreme poverty (GDP per capita excluded). (DOCX)

S5 Table The links between external debt, size of the tourism sector, and economic policy response to the COVID-19 pandemic: The influence of extreme poverty (GDP per capita and population over 65 excluded). (DOCX)

S1 Data (XLS)
