# Supplementary material for: COVID-19 economic stimulus packages, tourism industry and external debt: The influence of extreme poverty
Source: PLoS One. 2023 Aug 29;18(8):e0287384. doi: 10.1371/journal.pone.0287384 (PMC10464963; doi:10.1371/journal.pone.0287384)
Supplement: S1 Table — (DOCX) [file pone.0287384.s001.docx]

**Table S1: Summary Statistics**

| Variable | Less tourism-dependent economies | More tourism-dependent economies | Whole Sample |
| --- | --- | --- | --- |
| CESI | -0.48 | 0.47 | 2.02E-09 |
|  | (1.16) | (1.35) | (1.34) |
| Monetary policy index | -0.31 | 0.31 | 3.58E-09 |
|  | (0.97) | (1.22) | (1.14) |
| Ln Fiscal policy | 1.08 | 2.01 | 1.56 |
|  | (1.18) | (0.78) | (1.09) |
| Ln PVEXTD | 3.24 | 2.96 | 3.13 |
|  | (0.74) | (0.79) | (0.76) |
| Extreme poverty | 16.22 | 2.14 | 9.55 |
|  | (20.11) | (4.22) | (16.41) |
| Ln GDP per capita | 8.43 | 9.62 | 9.06 |
|  | (1.45) | (1.17) | (1.43) |
| Ln Hospital beds | 0.53 | 1.10 | 0.84 |
|  | (0.92) | (0.74) | (0.87) |
| Ln Fatality rate | -4.27 | -4.02 | -4.14 |
|  | (0.81) | (0.78) | (0.80) |
| Ln Health Expenditure | 1.78 | 1.91 | 1.85 |
|  | (0.36) | (0.41) | (0.39) |
| Population over 65 | 7.72 | 12.65 | 10.21 |
|  | (5.48) | (6.64) | (6.56) |

Notes: Ln denotes natural logarithm, PVEXTD is the present value of external debt (% of GNI), CESI is COVID-19 economic stimulus index, values in the parenthesis are standard deviation, and values without the parenthesis are mean. More tourism-dependent economies refer to the group of economies having the value of the ratio of tourist arrivals to total tourist arrivals for all countries covered in the sample that is greater than the median value of the ratio of tourist arrivals to total tourist arrivals for all countries covered in the sample, whereas less tourism-dependent economies have a ratio of tourist arrivals to total tourist arrivals that are less than the median value.
