# Supplementary material for: COVID-19 economic stimulus packages, tourism industry and external debt: The influence of extreme poverty
Source: PLoS One. 2023 Aug 29;18(8):e0287384. doi: 10.1371/journal.pone.0287384 (PMC10464963; doi:10.1371/journal.pone.0287384)
Supplement: S2 Table — (DOCX) [file pone.0287384.s002.docx]

**Table S2: Correlation Matrix**

|  | CESI | Ln PVEXTD | TODUM | EXTPOV | Ln GDPK | Ln HOSB | Ln FARATE | Ln HEALTHEXP | POP65 |
| --- | --- | --- | --- | --- | --- | --- | --- | --- | --- |
| CESI | 1 |  |  |  |  |  |  |  |  |
| Ln PVEXTD | -0.28 | 1 |  |  |  |  |  |  |  |
| TODUM | 0.47 | -0.11 | 1 |  |  |  |  |  |  |
| EXTPOV | -0.34 | 0.12 | -0.48 | 1 |  |  |  |  |  |
| Ln GDPK | 0.33 | -0.12 | 0.57 | -0.72 | 1 |  |  |  |  |
| Ln HOSB | 0.14 | 0.03 | 0.42 | -0.55 | 0.67 | 1 |  |  |  |
| Ln FARATE | -0.06 | -0.13 | 0.19 | -0.16 | 0.21 | 0.026 | 1 |  |  |
| Ln HEALTHEXP | -0.06 | 0.16 | -0.10 | -0.23 | 0.27 | 0.290 | 0.23 | 1 |  |
| POP65 | 0.12 | -0.07 | 0.43 | -0.59 | 0.64 | 0.68 | 0.20 | 0.40 | 1 |

Notes: Ln denotes natural logarithm, CESI is COVID-19 economic stimulus index, PVEXTD is the present value of external debt (% of GNI), TODUM is tourism-dependent dummy, EXTPOV is extreme poverty, GDPK is GDP per capita, HOSB is Hospital beds (per 1000 people), FARATE is fatality rate, HEALTHEXP refers to current health expenditure (% of GDP), POP65 is the percentage of population above 65.
