# Supplementary material for: COVID-19 economic stimulus packages, tourism industry and external debt: The influence of extreme poverty
Source: PLoS One. 2023 Aug 29;18(8):e0287384. doi: 10.1371/journal.pone.0287384 (PMC10464963; doi:10.1371/journal.pone.0287384)
Supplement: S3 Table — (DOCX) [file pone.0287384.s003.docx]

**Table S3: Multicollinearity tests**

| Dependent variable: CESI | | Dependent variable: Ln Fiscal Policy | |
| --- | --- | --- | --- |
| Variable | VIF | Variable | VIF |
| Ln GDPK | 3.14 | Ln GDPK | 3.14 |
| POP65 | 2.47 | POP65 | 2.58 |
| Ln HOSB | 2.39 | Ln HOSB | 2.43 |
| EXTPOV | 2.23 | EXTPOV | 2.32 |
| TODUM | 1.81 | TODUM | 1.84 |
| Ln HEALTHEXP | 1.54 | Ln HEALTHEXP | 1.55 |
| Ln FARATE | 1.20 | Ln FARATE | 1.19 |
| Ln PVEXTD | 1.11 | Ln PVEXTD | 1.17 |
| Mean VIF | 1.99 |  | 2.03 |

Notes: See Table 1 for definitions of notations. The estimates are similar if the monetary index is used as the dependent variable in lieu of CESI. The estimates are not reported but are available upon request.
