# Supplementary material for: COVID-19 economic stimulus packages, tourism industry and external debt: The influence of extreme poverty
Source: PLoS One. 2023 Aug 29;18(8):e0287384. doi: 10.1371/journal.pone.0287384 (PMC10464963; doi:10.1371/journal.pone.0287384)
Supplement: S4 Table — (DOCX) [file pone.0287384.s004.docx]

**Table S4: The links between external debt, size of the tourism sector, and economic policy response to the COVID-19 pandemic: the influence of extreme poverty (GDP per capita excluded)**

|  | DV: CESI | DV: Monetary Policy Index | DV: Ln Fiscal Policy | DV: CESI | DV: Monetary Policy Index | DV: Ln Fiscal Policy |
| --- | --- | --- | --- | --- | --- | --- |
| Variable | (1) | (2) | (3) | (4) | (5) | (6) |
| Ln PVEXTD | -0.185** | -0.227*** | -0.187* | -0.162* | -0.206** | -0.153 |
|  | (0.079) | (0.082) | (0.106) | (0.081) | (0.083) | (0.115) |
| TODUM | 0.556*** | 0.161 | 0.845*** | 0.416* | 0.029 | 0.685*** |
|  | (0.200) | (0.178) | (0.207) | (0.238) | (0.210) | (0.229) |
| EXTPOV | -0.007* | -0.014*** | 0.003 | -0.008* | -0.015*** | 0.002 |
|  | (0.004) | (0.005) | (0.007) | (0.004) | (0.005) | (0.007) |
| TODUM X EXTPOV |  |  |  | 0.028** | 0.026** | 0.033** |
|  |  |  |  | (0.012) | (0.012) | (0.016) |
| Ln HOSB | -0.057 | -0.080 | -0.005 | -0.045 | -0.069 | 0.006 |
|  | (0.102) | (0.093) | (0.144) | (0.100) | (0.092) | (0.142) |
| Ln FARATE | -0.166* | -0.042 | -0.367*** | -0.160* | -0.036 | -0.361*** |
|  | (0.094) | (0.086) | (0.129) | (0.092) | (0.086) | (0.124) |
| Ln HEALTHEXP | 0.220 | -0.082 | 0.440* | 0.164 | -0.135 | 0.370 |
|  | (0.336) | (0.340) | (0.243) | (0.352) | (0.350) | (0.239) |
| POP65 | -0.026 | -0.040* | -0.011 | -0.021 | -0.036 | -0.005 |
|  | (0.028) | (0.023) | (0.037) | (0.029) | (0.024) | (0.036) |
| Constant | -1.145 | 0.583 | -0.786 | -1.098 | 0.627 | -0.758 |
|  | (0.758) | (0.771) | (0.842) | (0.752) | (0.766) | (0.834) |
| Observations | 54 | 54 | 52 | 54 | 54 | 52 |
| R-Squared | 0.358 | 0.325 | 0.370 | 0.388 | 0.351 | 0.395 |

Notes: See Table 1 for definitions of notations. DV denotes the dependent variable. Robust standard errors in parentheses, levels of statistical significance indicated as *** p < 0.01, ** p < 0.05, * p < 0.10.
