# Supplementary material for: COVID-19 economic stimulus packages, tourism industry and external debt: The influence of extreme poverty
Source: PLoS One. 2023 Aug 29;18(8):e0287384. doi: 10.1371/journal.pone.0287384 (PMC10464963; doi:10.1371/journal.pone.0287384)
Supplement: S5 Table — (DOCX) [file pone.0287384.s005.docx]

**Table S5: The links between external debt, size of the tourism sector, and economic policy response to the COVID-19 pandemic: the influence of extreme poverty (GDP per capita and population over 65 excluded)**

|  | DV: CESI | DV: Monetary Policy Index | DV: Ln Fiscal Policy | DV: CESI | DV: Monetary Policy Index | DV: Ln Fiscal Policy |
| --- | --- | --- | --- | --- | --- | --- |
| Variable | (1) | (2) | (3) | (4) | (5) | (6) |
| Ln PVEXTD | -0.174** | -0.211** | -0.178* | -0.152* | -0.189** | -0.148 |
|  | (0.082) | (0.084) | (0.102) | (0.084) | (0.086) | (0.111) |
| TODUM | 0.522** | 0.107 | 0.835*** | 0.377 | -0.037 | 0.678*** |
|  | (0.201) | (0.182) | (0.215) | (0.236) | (0.210) | (0.241) |
| EXTPOV | -0.006 | -0.012** | 0.004 | -0.007* | -0.014** | 0.002 |
|  | (0.004) | (0.005) | (0.006) | (0.004) | (0.005) | (0.006) |
| TODUM X EXTPOV |  |  |  | 0.030** | 0.030*** | 0.033** |
|  |  |  |  | (0.012) | (0.010) | (0.016) |
| Ln HOSB | -0.108 | -0.159* | -0.027 | -0.085 | -0.137 | -0.003 |
|  | (0.093) | (0.091) | (0.113) | (0.092) | (0.090) | (0.113) |
| Ln FARATE | -0.175* | -0.056 | -0.371*** | -0.166* | -0.048 | -0.362*** |
|  | (0.095) | (0.084) | (0.127) | (0.092) | (0.084) | (0.121) |
| Ln HEALTHEXP | 0.144 | -0.202 | 0.405 | 0.098 | -0.248 | 0.354 |
|  | (0.317) | (0.316) | (0.251) | (0.327) | (0.321) | (0.249) |
| Constant | -1.240 | 0.434 | -0.840 | -1.171 | 0.503 | -0.781 |
|  | (0.742) | (0.786) | (0.775) | (0.743) | (0.784) | (0.766) |
| Observations | 54 | 54 | 52 | 54 | 54 | 52 |
| R-Squared | 0.342 | 0.286 | 0.368 | 0.377 | 0.320 | 0.395 |

Notes: See Table 1 for definitions of notations. DV denotes the dependent variable. Robust standard errors in parentheses, levels of statistical significance indicated as *** p < 0.01, ** p < 0.05, * p < 0.10.
